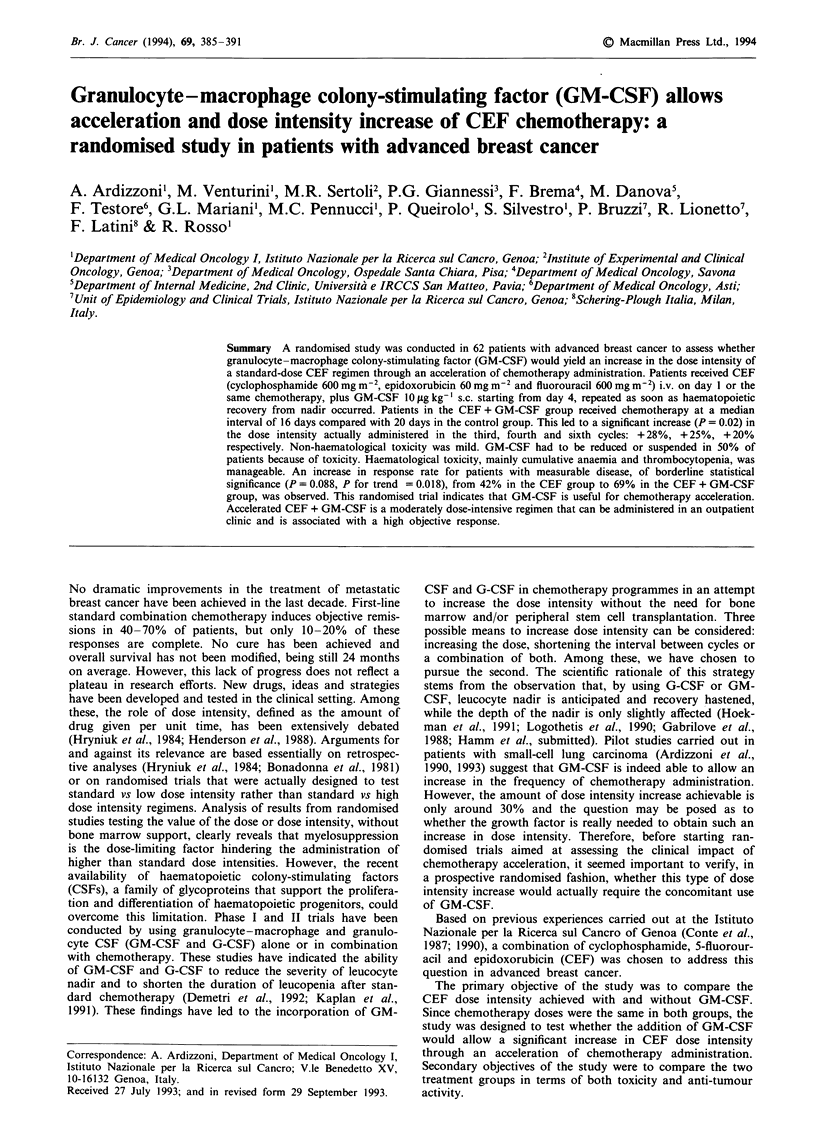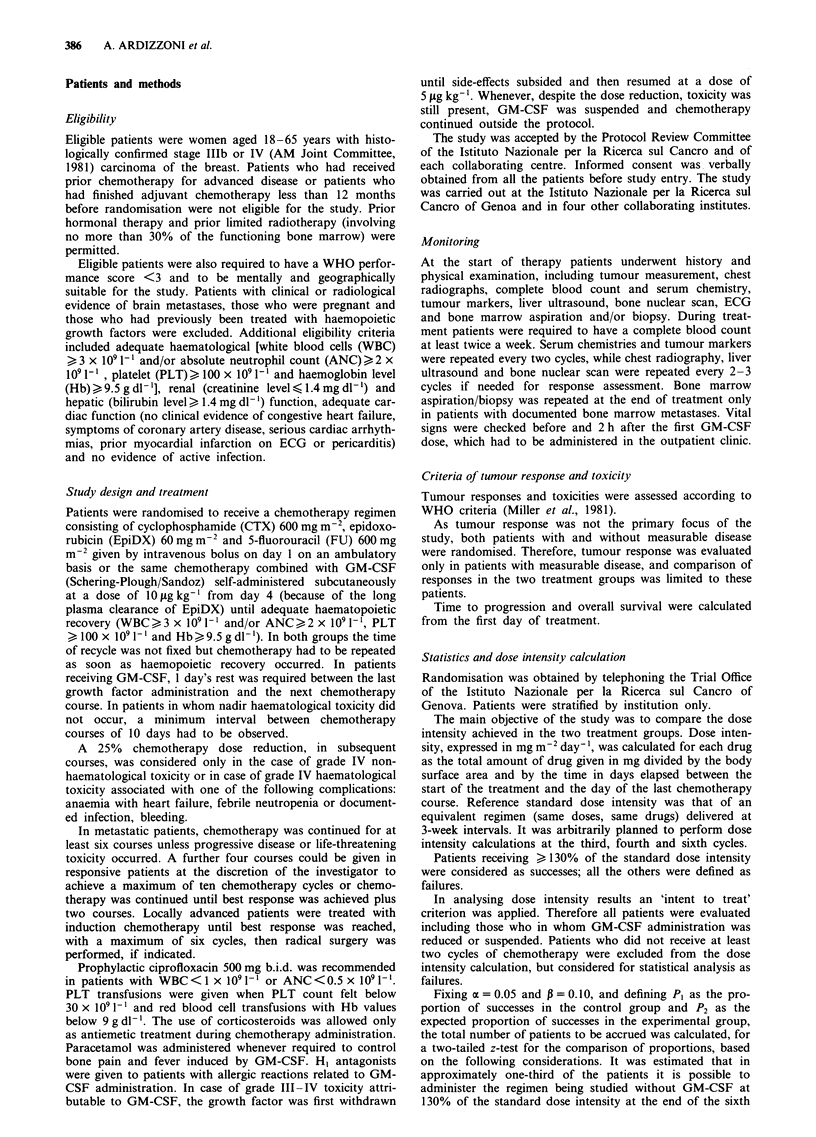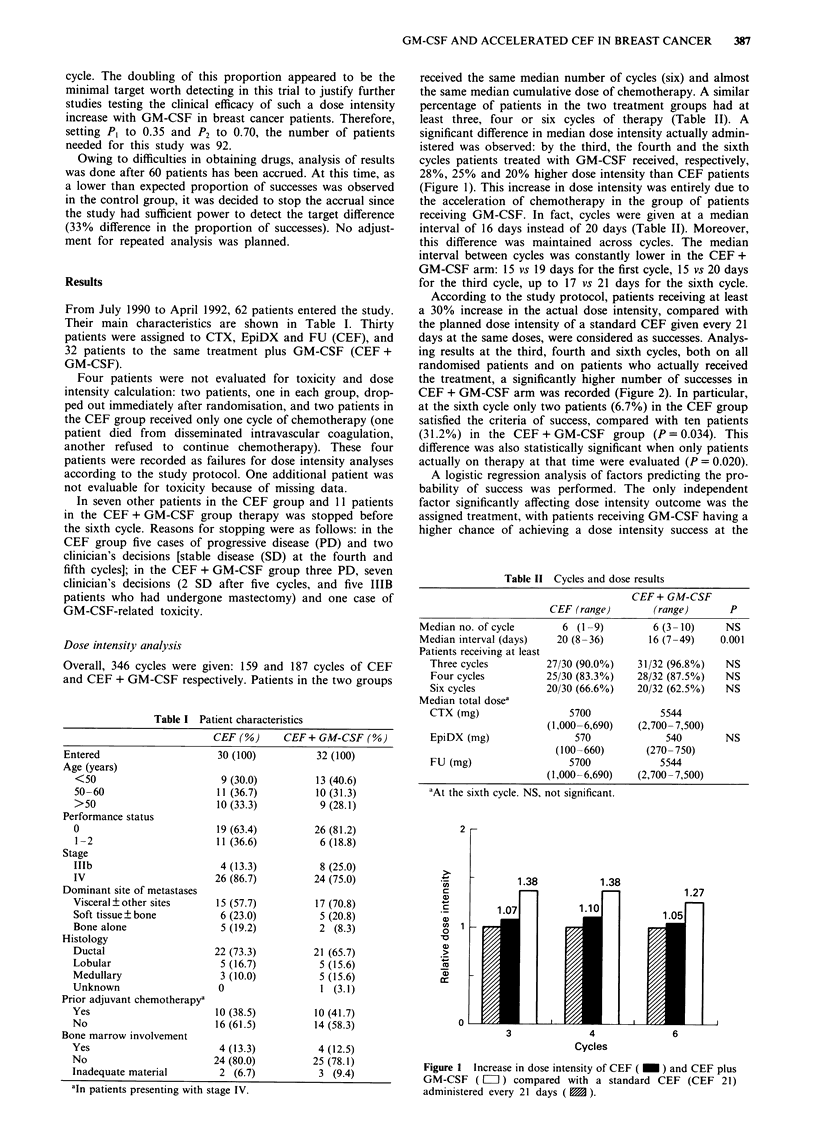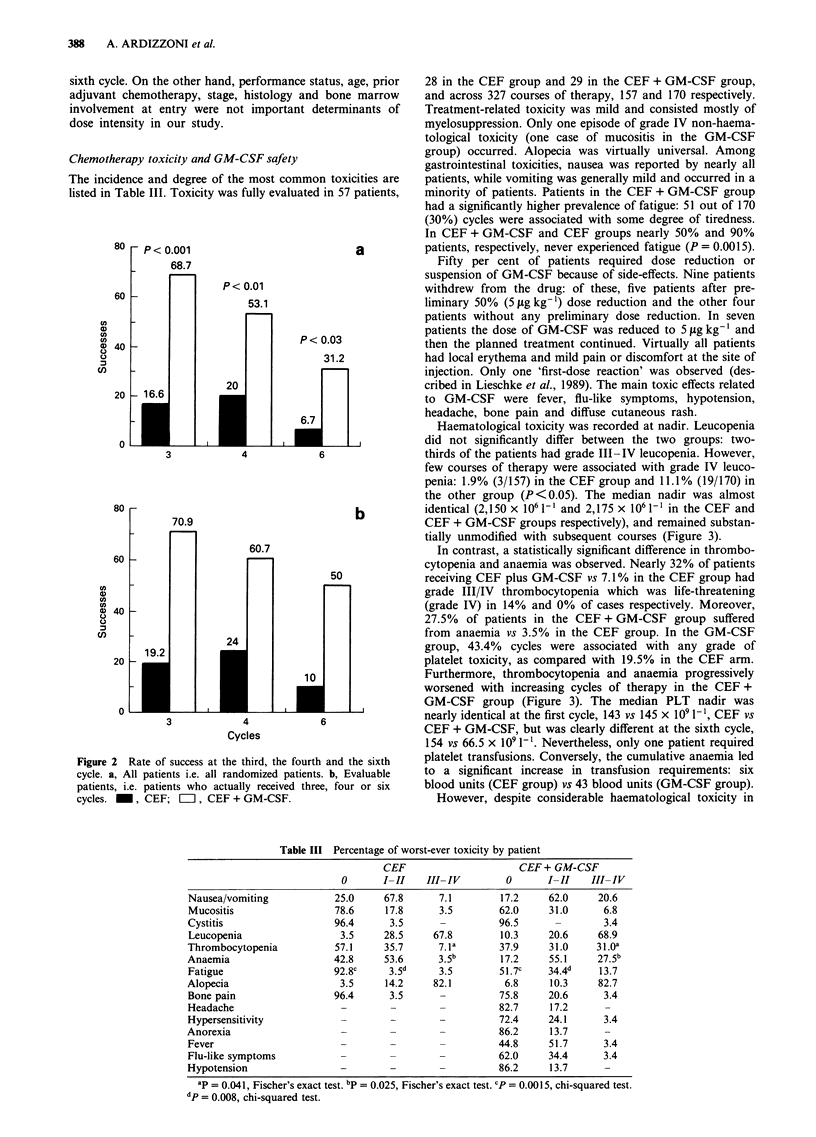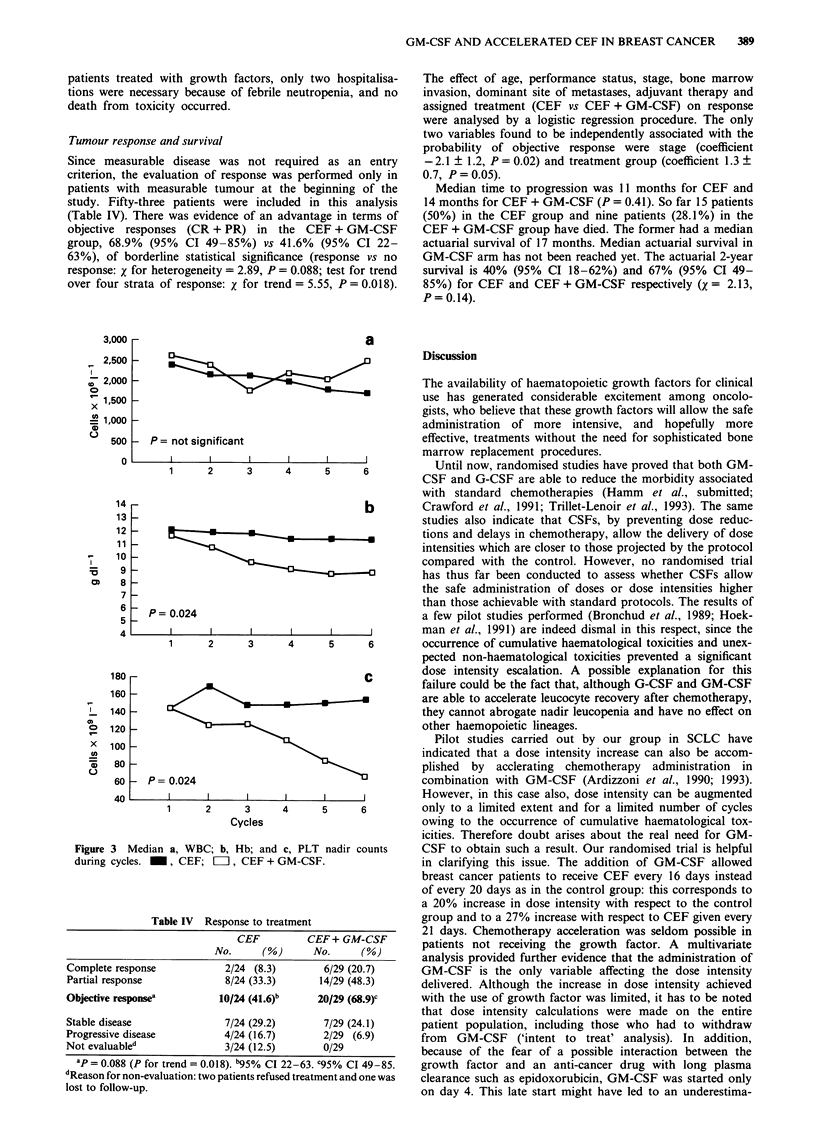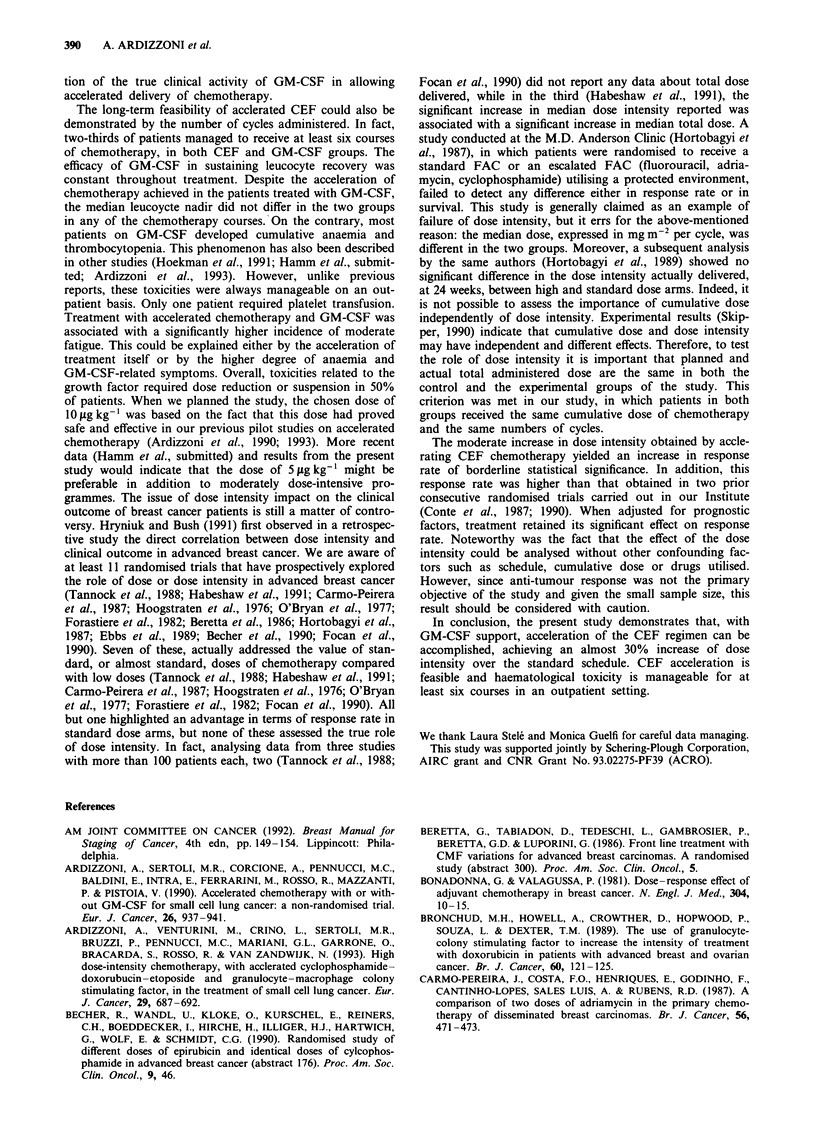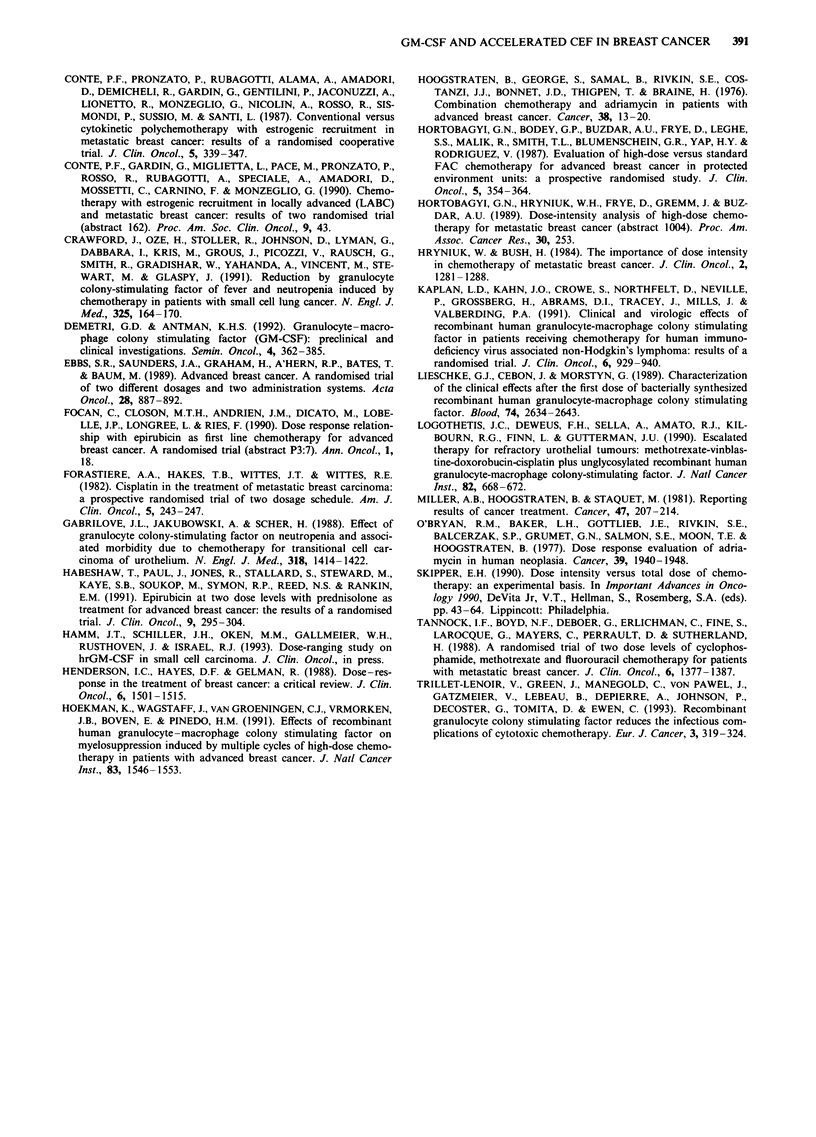# Granulocyte-macrophage colony-stimulating factor (GM-CSF) allows acceleration and dose intensity increase of CEF chemotherapy: a randomised study in patients with advanced breast cancer.

**DOI:** 10.1038/bjc.1994.71

**Published:** 1994-02

**Authors:** A. Ardizzoni, M. Venturini, M. R. Sertoli, P. G. Giannessi, F. Brema, M. Danova, F. Testore, G. L. Mariani, M. C. Pennucci, P. Queirolo

**Affiliations:** Department of Medical Oncology I, Istituto Nazionale per la Ricerca sul Cancro, Genoa, Italy.

## Abstract

A randomised study was conducted in 62 patients with advanced breast cancer to assess whether granulocyte-macrophage colony-stimulating factor (GM-CSF) would yield an increase in the dose intensity of a standard-dose CEF regimen through an acceleration of chemotherapy administration. Patients received CEF (cyclophosphamide 600 mg m-2, epidoxorubicin 60 mg m-2 and fluorouracil 600 mg m-2) i.v. on day 1 or the same chemotherapy, plus GM-CSF 10 micrograms kg-1 s.c. starting from day 4, repeated as soon as haematopoietic recovery from nadir occurred. Patients in the CEF + GM-CSF group received chemotherapy at a median interval of 16 days compared with 20 days in the control group. This led to a significant increase (P = 0.02) in the dose intensity actually administered in the third, fourth and sixth cycles: +28%, +25%, +20% respectively. Non-haematological toxicity was mild. GM-CSF had to be reduced or suspended in 50% of patients because of toxicity. Haematological toxicity, mainly cumulative anaemia and thrombocytopenia, was manageable. An increase in response rate for patients with measurable disease, of borderline statistical significance (P = 0.088, P for trend = 0.018), from 42% in the CEF group to 69% in the CEF + GM-CSF group, was observed. This randomised trial indicates that GM-CSF is useful for chemotherapy acceleration. Accelerated CEF + GM-CSF is a moderately dose-intensive regimen that can be administered in an outpatient clinic and is associated with a high objective response.


*Br. J. Cancer* (1994), **69**, 385–391 © Macmillan Press Ltd., 1994

### Granulocyte–macrophage colony-stimulating factor (GM-CSF) allows acceleration and dose intensity increase of CEF chemotherapy: a randomised study in patients with advanced breast cancer

A. Ardizzoni[1], M. Venturini[1], M.R. Sertoli[2], P.G. Giannessi[3], F. Brema[4], M. Danova[5], F. Testore[6], G.L. Mariani[1], M.C. Pennucci[1], P. Queirolo[1], S. Silvestro[1], P. Bruzzi[7], R. Lionetto[7], F. Latini[8] & R. Rosso[1]

[1]*Department of Medical Oncology I, Istituto Nazionale per la Ricerca sul Cancro, Genoa;* [2]*Institute of Experimental and Clinical Oncology, Genoa;* [3]*Department of Medical Oncology, Ospedale Santa Chiara, Pisa;* [4]*Department of Medical Oncology, Savona* [5]*Department of Internal Medicine, 2nd Clinic, Università e IRCCS San Matteo, Pavia;* [6]*Department of Medical Oncology, Asti;* [7]*Unit of Epidemiology and Clinical Trials, Istituto Nazionale per la Ricerca sul Cancro, Genoa;* [8]*Schering-Plough Italia, Milan, Italy.*

**Summary** A randomised study was conducted in 62 patients with advanced breast cancer to assess whether granulocyte–macrophage colony-stimulating factor (GM-CSF) would yield an increase in the dose intensity of a standard-dose CEF regimen through an acceleration of chemotherapy administration. Patients received CEF (cyclophosphamide 600 mg $m^{-2}$, epidoxorubicin 60 mg $m^{-2}$ and fluorouracil 600 mg $m^{-2}$) i.v. on day 1 or the same chemotherapy, plus GM-CSF 10 $\mu g\ kg^{-1}$ s.c. starting from day 4, repeated as soon as haematopoietic recovery from nadir occurred. Patients in the CEF + GM-CSF group received chemotherapy at a median interval of 16 days compared with 20 days in the control group. This led to a significant increase ($P = 0.02$) in the dose intensity actually administered in the third, fourth and sixth cycles: +28%, +25%, +20% respectively. Non-haematological toxicity was mild. GM-CSF had to be reduced or suspended in 50% of patients because of toxicity. Haematological toxicity, mainly cumulative anaemia and thrombocytopenia, was manageable. An increase in response rate for patients with measurable disease, of borderline statistical significance ($P = 0.088$, $P$ for trend $= 0.018$), from 42% in the CEF group to 69% in the CEF + GM-CSF group, was observed. This randomised trial indicates that GM-CSF is useful for chemotherapy acceleration. Accelerated CEF + GM-CSF is a moderately dose-intensive regimen that can be administered in an outpatient clinic and is associated with a high objective response.

No dramatic improvements in the treatment of metastatic breast cancer have been achieved in the last decade. First-line standard combination chemotherapy induces objective remissions in 40–70% of patients, but only 10–20% of these responses are complete. No cure has been achieved and overall survival has not been modified, being still 24 months on average. However, this lack of progress does not reflect a plateau in research efforts. New drugs, ideas and strategies have been developed and tested in the clinical setting. Among these, the role of dose intensity, defined as the amount of drug given per unit time, has been extensively debated (Hryniuk *et al.*, 1984; Henderson *et al.*, 1988). Arguments for and against its relevance are based essentially on retrospective analyses (Hryniuk *et al.*, 1984; Bonadonna *et al.*, 1981) or on randomised trials that were actually designed to test standard *vs* low dose intensity rather than standard *vs* high dose intensity regimens. Analysis of results from randomised studies testing the value of the dose or dose intensity, without bone marrow support, clearly reveals that myelosuppression is the dose-limiting factor hindering the administration of higher than standard dose intensities. However, the recent availability of haematopoietic colony-stimulating factors (CSFs), a family of glycoproteins that support the proliferation and differentiation of haematopoietic progenitors, could overcome this limitation. Phase I and II trials have been conducted by using granulocyte–macrophage and granulocyte CSF (GM-CSF and G-CSF) alone or in combination with chemotherapy. These studies have indicated the ability of GM-CSF and G-CSF to reduce the severity of leucocyte nadir and to shorten the duration of leucopenia after standard chemotherapy (Demetri *et al.*, 1992; Kaplan *et al.*, 1991). These findings have led to the incorporation of GM-CSF and G-CSF in chemotherapy programmes in an attempt to increase the dose intensity without the need for bone marrow and/or peripheral stem cell transplantation. Three possible means to increase dose intensity can be considered: increasing the dose, shortening the interval between cycles or a combination of both. Among these, we have chosen to pursue the second. The scientific rationale of this strategy stems from the observation that, by using G-CSF or GM-CSF, leucocyte nadir is anticipated and recovery hastened, while the depth of the nadir is only slightly affected (Hoekman *et al.*, 1991; Logothetis *et al.*, 1990; Gabrilove *et al.*, 1988; Hamm *et al.*, submitted). Pilot studies carried out in patients with small-cell lung carcinoma (Ardizzoni *et al.*, 1990, 1993) suggest that GM-CSF is indeed able to allow an increase in the frequency of chemotherapy administration. However, the amount of dose intensity increase achievable is only around 30% and the question may be posed as to whether the growth factor is really needed to obtain such an increase in dose intensity. Therefore, before starting randomised trials aimed at assessing the clinical impact of chemotherapy acceleration, it seemed important to verify, in a prospective randomised fashion, whether this type of dose intensity increase would actually require the concomitant use of GM-CSF.

Based on previous experiences carried out at the Istituto Nazionale per la Ricerca sul Cancro of Genoa (Conte *et al.*, 1987; 1990), a combination of cyclophosphamide, 5-fluorouracil and epidoxorubicin (CEF) was chosen to address this question in advanced breast cancer.

The primary objective of the study was to compare the CEF dose intensity achieved with and without GM-CSF. Since chemotherapy doses were the same in both groups, the study was designed to test whether the addition of GM-CSF would allow a significant increase in CEF dose intensity through an acceleration of chemotherapy administration. Secondary objectives of the study were to compare the two treatment groups in terms of both toxicity and anti-tumour activity.

Correspondence: A. Ardizzoni, Department of Medical Oncology I, Istituto Nazionale per la Ricerca sul Cancro; V.le Benedetto XV, 10-16132 Genoa, Italy.
Received 27 July 1993; and in revised form 29 September 1993.

### Patients and methods

### *Eligibility*

Eligible patients were women aged 18–65 years with histologically confirmed stage IIIb or IV (AM Joint Committee, 1981) carcinoma of the breast. Patients who had received prior chemotherapy for advanced disease or patients who had finished adjuvant chemotherapy less than 12 months before randomisation were not eligible for the study. Prior hormonal therapy and prior limited radiotherapy (involving no more than 30% of the functioning bone marrow) were permitted.

Eligible patients were also required to have a WHO performance score <3 and to be mentally and geographically suitable for the study. Patients with clinical or radiological evidence of brain metastases, those who were pregnant and those who had previously been treated with haemopoietic growth factors were excluded. Additional eligibility criteria included adequate haematological [white blood cells (WBC) $\geqslant 3 \times 10^9 l^{-1}$ and/or absolute neutrophil count (ANC) $\geqslant 2 \times 10^9 l^{-1}$, platelet (PLT) $\geqslant 100 \times 10^9 l^{-1}$ and haemoglobin level (Hb) $\geqslant 9.5$ g $dl^{-1}$], renal (creatinine level $\leqslant 1.4$ mg $dl^{-1}$) and hepatic (bilirubin level $\geqslant 1.4$ mg $dl^{-1}$) function, adequate cardiac function (no clinical evidence of congestive heart failure, symptoms of coronary artery disease, serious cardiac arrhythmias, prior myocardial infarction on ECG or pericarditis) and no evidence of active infection.

### *Study design and treatment*

Patients were randomised to receive a chemotherapy regimen consisting of cyclophosphamide (CTX) 600 mg $m^{-2}$, epidoxorubicin (EpiDX) 60 mg $m^{-2}$ and 5-fluorouracil (FU) 600 mg $m^{-2}$ given by intravenous bolus on day 1 on an ambulatory basis or the same chemotherapy combined with GM-CSF (Schering-Plough/Sandoz) self-administered subcutaneously at a dose of 10 $\mu g$ $kg^{-1}$ from day 4 (because of the long plasma clearance of EpiDX) until adequate haematopoietic recovery (WBC $\geqslant 3 \times 10^9 l^{-1}$ and/or ANC $\geqslant 2 \times 10^9 l^{-1}$, PLT $\geqslant 100 \times 10^9 l^{-1}$ and Hb $\geqslant 9.5$ g $dl^{-1}$). In both groups the time of recycle was not fixed but chemotherapy had to be repeated as soon as haemopoietic recovery occurred. In patients receiving GM-CSF, 1 day's rest was required between the last growth factor administration and the next chemotherapy course. In patients in whom nadir haematological toxicity did not occur, a minimum interval between chemotherapy courses of 10 days had to be observed.

A 25% chemotherapy dose reduction, in subsequent courses, was considered only in the case of grade IV non-haematological toxicity or in case of grade IV haematological toxicity associated with one of the following complications: anaemia with heart failure, febrile neutropenia or documented infection, bleeding.

In metastatic patients, chemotherapy was continued for at least six courses unless progressive disease or life-threatening toxicity occurred. A further four courses could be given in responsive patients at the discretion of the investigator to achieve a maximum of ten chemotherapy cycles or chemotherapy was continued until best response was achieved plus two courses. Locally advanced patients were treated with induction chemotherapy until best response was reached, with a maximum of six cycles, then radical surgery was performed, if indicated.

Prophylactic ciprofloxacin 500 mg b.i.d. was recommended in patients with WBC $< 1 \times 10^9 l^{-1}$ or ANC $< 0.5 \times 10^9 l^{-1}$. PLT transfusions were given when PLT count felt below $30 \times 10^9 l^{-1}$ and red blood cell transfusions with Hb values below 9 g $dl^{-1}$. The use of corticosteroids was allowed only as antiemetic treatment during chemotherapy administration. Paracetamol was administered whenever required to control bone pain and fever induced by GM-CSF. $H_1$ antagonists were given to patients with allergic reactions related to GM-CSF administration. In case of grade III–IV toxicity attributable to GM-CSF, the growth factor was first withdrawn until side-effects subsided and then resumed at a dose of 5 $\mu g$ $kg^{-1}$. Whenever, despite the dose reduction, toxicity was still present, GM-CSF was suspended and chemotherapy continued outside the protocol.

The study was accepted by the Protocol Review Committee of the Istituto Nazionale per la Ricerca sul Cancro and of each collaborating centre. Informed consent was verbally obtained from all the patients before study entry. The study was carried out at the Istituto Nazionale per la Ricerca sul Cancro of Genoa and in four other collaborating institutes.

### *Monitoring*

At the start of therapy patients underwent history and physical examination, including tumour measurement, chest radiographs, complete blood count and serum chemistry, tumour markers, liver ultrasound, bone nuclear scan, ECG and bone marrow aspiration and/or biopsy. During treatment patients were required to have a complete blood count at least twice a week. Serum chemistries and tumour markers were repeated every two cycles, while chest radiography, liver ultrasound and bone nuclear scan were repeated every 2–3 cycles if needed for response assessment. Bone marrow aspiration/biopsy was repeated at the end of treatment only in patients with documented bone marrow metastases. Vital signs were checked before and 2 h after the first GM-CSF dose, which had to be administered in the outpatient clinic.

### *Criteria of tumour response and toxicity*

Tumour responses and toxicities were assessed according to WHO criteria (Miller *et al.*, 1981).

As tumour response was not the primary focus of the study, both patients with and without measurable disease were randomised. Therefore, tumour response was evaluated only in patients with measurable disease, and comparison of responses in the two treatment groups was limited to these patients.

Time to progression and overall survival were calculated from the first day of treatment.

### *Statistics and dose intensity calculation*

Randomisation was obtained by telephoning the Trial Office of the Istituto Nazionale per la Ricerca sul Cancro of Genova. Patients were stratified by institution only.

The main objective of the study was to compare the dose intensity achieved in the two treatment groups. Dose intensity, expressed in mg $m^{-2}$ $day^{-1}$, was calculated for each drug as the total amount of drug given in mg divided by the body surface area and by the time in days elapsed between the start of the treatment and the day of the last chemotherapy course. Reference standard dose intensity was that of an equivalent regimen (same doses, same drugs) delivered at 3-week intervals. It was arbitrarily planned to perform dose intensity calculations at the third, fourth and sixth cycles.

Patients receiving $\geqslant 130\%$ of the standard dose intensity were considered as successes; all the others were defined as failures.

In analysing dose intensity results an 'intent to treat' criterion was applied. Therefore all patients were evaluated including those who in whom GM-CSF administration was reduced or suspended. Patients who did not receive at least two cycles of chemotherapy were excluded from the dose intensity calculation, but considered for statistical analysis as failures.

Fixing $\alpha = 0.05$ and $\beta = 0.10$, and defining $P_1$ as the proportion of successes in the control group and $P_2$ as the expected proportion of successes in the experimental group, the total number of patients to be accrued was calculated, for a two-tailed $z$-test for the comparison of proportions, based on the following considerations. It was estimated that in approximately one-third of the patients it is possible to administer the regimen being studied without GM-CSF at 130% of the standard dose intensity at the end of the sixth cycle. The doubling of this proportion appeared to be the minimal target worth detecting in this trial to justify further studies testing the clinical efficacy of such a dose intensity increase with GM-CSF in breast cancer patients. Therefore, setting $P_1$ to 0.35 and $P_2$ to 0.70, the number of patients needed for this study was 92.

Owing to difficulties in obtaining drugs, analysis of results was done after 60 patients has been accrued. At this time, as a lower than expected proportion of successes was observed in the control group, it was decided to stop the accrual since the study had sufficient power to detect the target difference (33% difference in the proportion of successes). No adjustment for repeated analysis was planned.

### Results

From July 1990 to April 1992, 62 patients entered the study. Their main characteristics are shown in Table I. Thirty patients were assigned to CTX, EpiDX and FU (CEF), and 32 patients to the same treatment plus GM-CSF (CEF + GM-CSF).

Four patients were not evaluated for toxicity and dose intensity calculation: two patients, one in each group, dropped out immediately after randomisation, and two patients in the CEF group received only one cycle of chemotherapy (one patient died from disseminated intravascular coagulation, another refused to continue chemotherapy). These four patients were recorded as failures for dose intensity analyses according to the study protocol. One additional patient was not evaluable for toxicity because of missing data.

In seven other patients in the CEF group and 11 patients in the CEF + GM-CSF group therapy was stopped before the sixth cycle. Reasons for stopping were as follows: in the CEF group five cases of progressive disease (PD) and two clinician's decisions [stable disease (SD) at the fourth and fifth cycles]; in the CEF + GM-CSF group three PD, seven clinician's decisions (2 SD after five cycles, and five IIIB patients who had undergone mastectomy) and one case of GM-CSF-related toxicity.

### *Dose intensity analysis*

Overall, 346 cycles were given: 159 and 187 cycles of CEF and CEF + GM-CSF respectively. Patients in the two groups received the same median number of cycles (six) and almost the same median cumulative dose of chemotherapy. A similar percentage of patients in the two treatment groups had at least three, four or six cycles of therapy (Table II). A significant difference in median dose intensity actually administered was observed: by the third, the fourth and the sixth cycles patients treated with GM-CSF received, respectively, 28%, 25% and 20% higher dose intensity than CEF patients (Figure 1). This increase in dose intensity was entirely due to the acceleration of chemotherapy in the group of patients receiving GM-CSF. In fact, cycles were given at a median interval of 16 days instead of 20 days (Table II). Moreover, this difference was maintained across cycles. The median interval between cycles was constantly lower in the CEF + GM-CSF arm: 15 *vs* 19 days for the first cycle, 15 *vs* 20 days for the third cycle, up to 17 *vs* 21 days for the sixth cycle.

According to the study protocol, patients receiving at least a 30% increase in the actual dose intensity, compared with the planned dose intensity of a standard CEF given every 21 days at the same doses, were considered as successes. Analysing results at the third, fourth and sixth cycles, both on all randomised patients and on patients who actually received the treatment, a significantly higher number of successes in CEF + GM-CSF arm was recorded (Figure 2). In particular, at the sixth cycle only two patients (6.7%) in the CEF group satisfied the criteria of success, compared with ten patients (31.2%) in the CEF + GM-CSF group ($P = 0.034$). This difference was also statistically significant when only patients actually on therapy at that time were evaluated ($P = 0.020$).

A logistic regression analysis of factors predicting the probability of success was performed. The only independent factor significantly affecting dose intensity outcome was the assigned treatment, with patients receiving GM-CSF having a higher chance of achieving a dose intensity success at the

**Table I** Patient characteristics

| | *CEF (%)* | *CEF + GM-CSF (%)* |
|---|---|---|
| Entered | 30 (100) | 32 (100) |
| Age (years) | | |
| <50 | 9 (30.0) | 13 (40.6) |
| 50-60 | 11 (36.7) | 10 (31.3) |
| >50 | 10 (33.3) | 9 (28.1) |
| Performance status | | |
| 0 | 19 (63.4) | 26 (81.2) |
| 1-2 | 11 (36.6) | 6 (18.8) |
| Stage | | |
| IIIb | 4 (13.3) | 8 (25.0) |
| IV | 26 (86.7) | 24 (75.0) |
| Dominant site of metastases | | |
| Visceral ± other sites | 15 (57.7) | 17 (70.8) |
| Soft tissue ± bone | 6 (23.0) | 5 (20.8) |
| Bone alone | 5 (19.2) | 2 (8.3) |
| Histology | | |
| Ductal | 22 (73.3) | 21 (65.7) |
| Lobular | 5 (16.7) | 5 (15.6) |
| Medullary | 3 (10.0) | 5 (15.6) |
| Unknown | 0 | 1 (3.1) |
| Prior adjuvant chemotherapy[a] | | |
| Yes | 10 (38.5) | 10 (41.7) |
| No | 16 (61.5) | 14 (58.3) |
| Bone marrow involvement | | |
| Yes | 4 (13.3) | 4 (12.5) |
| No | 24 (80.0) | 25 (78.1) |
| Inadequate material | 2 (6.7) | 3 (9.4) |

[a]In patients presenting with stage IV.

**Table II** Cycles and dose results

| | *CEF (range)* | *CEF + GM-CSF (range)* | *P* |
|---|---|---|---|
| Median no. of cycle | 6 (1-9) | 6 (3-10) | NS |
| Median interval (days) | 20 (8-36) | 16 (7-49) | 0.001 |
| Patients receiving at least | | | |
| Three cycles | 27/30 (90.0%) | 31/32 (96.8%) | NS |
| Four cycles | 25/30 (83.3%) | 28/32 (87.5%) | NS |
| Six cycles | 20/30 (66.6%) | 20/32 (62.5%) | NS |
| Median total dose[a] | | | |
| CTX (mg) | 5700 (1,000-6,690) | 5544 (2,700-7,500) | |
| EpiDX (mg) | 570 (100-660) | 540 (270-750) | NS |
| FU (mg) | 5700 (1,000-6,690) | 5544 (2,700-7,500) | |

[a]At the sixth cycle. NS, not significant.

**Figure 1** Increase in dose intensity of CEF (■) and CEF plus GM-CSF (□) compared with a standard CEF (CEF 21) administered every 21 days (▨).

sixth cycle. On the other hand, performance status, age, prior adjuvant chemotherapy, stage, histology and bone marrow involvement at entry were not important determinants of dose intensity in our study.

*Chemotherapy toxicity and GM-CSF safety*

The incidence and degree of the most common toxicities are listed in Table III. Toxicity was fully evaluated in 57 patients,

**Figure 2** Rate of success at the third, the fourth and the sixth cycle. **a**, All patients i.e. all randomized patients. **b**, Evaluable patients, i.e. patients who actually received three, four or six cycles. ■, CEF; □, CEF + GM-CSF.

28 in the CEF group and 29 in the CEF + GM-CSF group, and across 327 courses of therapy, 157 and 170 respectively. Treatment-related toxicity was mild and consisted mostly of myelosuppression. Only one episode of grade IV non-haematological toxicity (one case of mucositis in the GM-CSF group) occurred. Alopecia was virtually universal. Among gastrointestinal toxicities, nausea was reported by nearly all patients, while vomiting was generally mild and occurred in a minority of patients. Patients in the CEF + GM-CSF group had a significantly higher prevalence of fatigue: 51 out of 170 (30%) cycles were associated with some degree of tiredness. In CEF + GM-CSF and CEF groups nearly 50% and 90% patients, respectively, never experienced fatigue ($P = 0.0015$).

Fifty per cent of patients required dose reduction or suspension of GM-CSF because of side-effects. Nine patients withdrew from the drug: of these, five patients after preliminary 50% ($5\,\mu g\,kg^{-1}$) dose reduction and the other four patients without any preliminary dose reduction. In seven patients the dose of GM-CSF was reduced to $5\,\mu g\,kg^{-1}$ and then the planned treatment continued. Virtually all patients had local erythema and mild pain or discomfort at the site of injection. Only one 'first-dose reaction' was observed (described in Lieschke *et al.*, 1989). The main toxic effects related to GM-CSF were fever, flu-like symptoms, hypotension, headache, bone pain and diffuse cutaneous rash.

Haematological toxicity was recorded at nadir. Leucopenia did not significantly differ between the two groups: two-thirds of the patients had grade III–IV leucopenia. However, few courses of therapy were associated with grade IV leucopenia: 1.9% (3/157) in the CEF group and 11.1% (19/170) in the other group ($P < 0.05$). The median nadir was almost identical ($2,150 \times 10^6\,l^{-1}$ and $2,175 \times 10^6\,l^{-1}$ in the CEF and CEF + GM-CSF groups respectively), and remained substantially unmodified with subsequent courses (Figure 3).

In contrast, a statistically significant difference in thrombocytopenia and anaemia was observed. Nearly 32% of patients receiving CEF plus GM-CSF *vs* 7.1% in the CEF group had grade III/IV thrombocytopenia which was life-threatening (grade IV) in 14% and 0% of cases respectively. Moreover, 27.5% of patients in the CEF + GM-CSF group suffered from anaemia *vs* 3.5% in the CEF group. In the GM-CSF group, 43.4% cycles were associated with any grade of platelet toxicity, as compared with 19.5% in the CEF arm. Furthermore, thrombocytopenia and anaemia progressively worsened with increasing cycles of therapy in the CEF + GM-CSF group (Figure 3). The median PLT nadir was nearly identical at the first cycle, 143 *vs* $145 \times 10^9\,l^{-1}$, CEF *vs* CEF + GM-CSF, but was clearly different at the sixth cycle, 154 *vs* $66.5 \times 10^9\,l^{-1}$. Nevertheless, only one patient required platelet transfusions. Conversely, the cumulative anaemia led to a significant increase in transfusion requirements: six blood units (CEF group) *vs* 43 blood units (GM-CSF group).

However, despite considerable haematological toxicity in

**Table III** Percentage of worst-ever toxicity by patient

| | *CEF* | | | *CEF + GM-CSF* | | |
|---|---|---|---|---|---|---|
| | *0* | *I–II* | *III–IV* | *0* | *I–II* | *III–IV* |
| Nausea/vomiting | 25.0 | 67.8 | 7.1 | 17.2 | 62.0 | 20.6 |
| Mucositis | 78.6 | 17.8 | 3.5 | 62.0 | 31.0 | 6.8 |
| Cystitis | 96.4 | 3.5 | – | 96.5 | – | 3.4 |
| Leucopenia | 3.5 | 28.5 | 67.8 | 10.3 | 20.6 | 68.9 |
| Thrombocytopenia | 57.1 | 35.7 | 7.1[a] | 37.9 | 31.0 | 31.0[a] |
| Anaemia | 42.8 | 53.6 | 3.5[b] | 17.2 | 55.1 | 27.5[b] |
| Fatigue | 92.8[c] | 3.5[d] | 3.5 | 51.7[c] | 34.4[d] | 13.7 |
| Alopecia | 3.5 | 14.2 | 82.1 | 6.8 | 10.3 | 82.7 |
| Bone pain | 96.4 | 3.5 | – | 75.8 | 20.6 | 3.4 |
| Headache | – | – | – | 82.7 | 17.2 | – |
| Hypersensitivity | – | – | – | 72.4 | 24.1 | 3.4 |
| Anorexia | – | – | – | 86.2 | 13.7 | – |
| Fever | – | – | – | 44.8 | 51.7 | 3.4 |
| Flu-like symptoms | – | – | – | 62.0 | 34.4 | 3.4 |
| Hypotension | – | – | – | 86.2 | 13.7 | – |

[a]$P = 0.041$, Fischer's exact test. [b]$P = 0.025$, Fischer's exact test. [c]$P = 0.0015$, chi-squared test. [d]$P = 0.008$, chi-squared test.

patients treated with growth factors, only two hospitalisations were necessary because of febrile neutropenia, and no death from toxicity occurred.

*Tumour response and survival*

Since measurable disease was not required as an entry criterion, the evaluation of response was performed only in patients with measurable tumour at the beginning of the study. Fifty-three patients were included in this analysis (Table IV). There was evidence of an advantage in terms of objective responses (CR + PR) in the CEF + GM-CSF group, 68.9% (95% CI 49–85%) *vs* 41.6% (95% CI 22–63%), of borderline statistical significance (response *vs* no response: $\chi$ for heterogeneity = 2.89, $P = 0.088$; test for trend over four strata of response: $\chi$ for trend = 5.55, $P = 0.018$).

**Figure 3** Median **a**, WBC; **b**, Hb; and **c**, PLT nadir counts during cycles. ■, CEF; □, CEF + GM-CSF.

**Table IV** Response to treatment

| | *CEF* No. | *CEF* (%) | *CEF + GM-CSF* No. | *CEF + GM-CSF* (%) |
|---|---|---|---|---|
| Complete response | 2/24 | (8.3) | 6/29 | (20.7) |
| Partial response | 8/24 | (33.3) | 14/29 | (48.3) |
| **Objective response**[a] | **10/24** | **(41.6)**[b] | **20/29** | **(68.9)**[c] |
| Stable disease | 7/24 | (29.2) | 7/29 | (24.1) |
| Progressive disease | 4/24 | (16.7) | 2/29 | (6.9) |
| Not evaluable[d] | 3/24 | (12.5) | 0/29 | |

[a]$P = 0.088$ ($P$ for trend = 0.018). [b]95% CI 22–63. [c]95% CI 49–85. [d]Reason for non-evaluation: two patients refused treatment and one was lost to follow-up.

The effect of age, performance status, stage, bone marrow invasion, dominant site of metastases, adjuvant therapy and assigned treatment (CEF *vs* CEF + GM-CSF) on response were analysed by a logistic regression procedure. The only two variables found to be independently associated with the probability of objective response were stage (coefficient $-2.1 \pm 1.2$, $P = 0.02$) and treatment group (coefficient $1.3 \pm 0.7$, $P = 0.05$).

Median time to progression was 11 months for CEF and 14 months for CEF + GM-CSF ($P = 0.41$). So far 15 patients (50%) in the CEF group and nine patients (28.1%) in the CEF + GM-CSF group have died. The former had a median actuarial survival of 17 months. Median actuarial survival in GM-CSF arm has not been reached yet. The actuarial 2-year survival is 40% (95% CI 18–62%) and 67% (95% CI 49–85%) for CEF and CEF + GM-CSF respectively ($\chi = 2.13$, $P = 0.14$).

### Discussion

The availability of haematopoietic growth factors for clinical use has generated considerable excitement among oncologists, who believe that these growth factors will allow the safe administration of more intensive, and hopefully more effective, treatments without the need for sophisticated bone marrow replacement procedures.

Until now, randomised studies have proved that both GM-CSF and G-CSF are able to reduce the morbidity associated with standard chemotherapies (Hamm *et al.*, submitted; Crawford *et al.*, 1991; Trillet-Lenoir *et al.*, 1993). The same studies also indicate that CSFs, by preventing dose reductions and delays in chemotherapy, allow the delivery of dose intensities which are closer to those projected by the protocol compared with the control. However, no randomised trial has thus far been conducted to assess whether CSFs allow the safe administration of doses or dose intensities higher than those achievable with standard protocols. The results of a few pilot studies performed (Bronchud *et al.*, 1989; Hoekman *et al.*, 1991) are indeed dismal in this respect, since the occurrence of cumulative haematological toxicities and unexpected non-haematological toxicities prevented a significant dose intensity escalation. A possible explanation for this failure could be the fact that, although G-CSF and GM-CSF are able to accelerate leucocyte recovery after chemotherapy, they cannot abrogate nadir leucopenia and have no effect on other haemopoietic lineages.

Pilot studies carried out by our group in SCLC have indicated that a dose intensity increase can also be accomplished by acclerating chemotherapy administration in combination with GM-CSF (Ardizzoni *et al.*, 1990; 1993). However, in this case also, dose intensity can be augmented only to a limited extent and for a limited number of cycles owing to the occurrence of cumulative haematological toxicities. Therefore doubt arises about the real need for GM-CSF to obtain such a result. Our randomised trial is helpful in clarifying this issue. The addition of GM-CSF allowed breast cancer patients to receive CEF every 16 days instead of every 20 days as in the control group: this corresponds to a 20% increase in dose intensity with respect to the control group and to a 27% increase with respect to CEF given every 21 days. Chemotherapy acceleration was seldom possible in patients not receiving the growth factor. A multivariate analysis provided further evidence that the administration of GM-CSF is the only variable affecting the dose intensity delivered. Although the increase in dose intensity achieved with the use of growth factor was limited, it has to be noted that dose intensity calculations were made on the entire patient population, including those who had to withdraw from GM-CSF ('intent to treat' analysis). In addition, because of the fear of a possible interaction between the growth factor and an anti-cancer drug with long plasma clearance such as epidoxorubicin, GM-CSF was started only on day 4. This late start might have led to an underestimation of the true clinical activity of GM-CSF in allowing accelerated delivery of chemotherapy.

The long-term feasibility of acclerated CEF could also be demonstrated by the number of cycles administered. In fact, two-thirds of patients managed to receive at least six courses of chemotherapy, in both CEF and GM-CSF groups. The efficacy of GM-CSF in sustaining leucocyte recovery was constant throughout treatment. Despite the acceleration of chemotherapy achieved in the patients treated with GM-CSF, the median leucoycte nadir did not differ in the two groups in any of the chemotherapy courses. On the contrary, most patients on GM-CSF developed cumulative anaemia and thrombocytopenia. This phenomenon has also been described in other studies (Hoekman *et al.*, 1991; Hamm *et al.*, submitted; Ardizzoni *et al.*, 1993). However, unlike previous reports, these toxicities were always manageable on an outpatient basis. Only one patient required platelet transfusion. Treatment with accelerated chemotherapy and GM-CSF was associated with a significantly higher incidence of moderate fatigue. This could be explained either by the acceleration of treatment itself or by the higher degree of anaemia and GM-CSF-related symptoms. Overall, toxicities related to the growth factor required dose reduction or suspension in 50% of patients. When we planned the study, the chosen dose of $10\,\mu g\,kg^{-1}$ was based on the fact that this dose had proved safe and effective in our previous pilot studies on accelerated chemotherapy (Ardizzoni *et al.*, 1990; 1993). More recent data (Hamm *et al.*, submitted) and results from the present study would indicate that the dose of $5\,\mu g\,kg^{-1}$ might be preferable in addition to moderately dose-intensive programmes. The issue of dose intensity impact on the clinical outcome of breast cancer patients is still a matter of controversy. Hryniuk and Bush (1991) first observed in a retrospective study the direct correlation between dose intensity and clinical outcome in advanced breast cancer. We are aware of at least 11 randomised trials that have prospectively explored the role of dose or dose intensity in advanced breast cancer (Tannock *et al.*, 1988; Habeshaw *et al.*, 1991; Carmo-Peirera *et al.*, 1987; Hoogstraten *et al.*, 1976; O'Bryan *et al.*, 1977; Forastiere *et al.*, 1982; Beretta *et al.*, 1986; Hortobagyi *et al.*, 1987; Ebbs *et al.*, 1989; Becher *et al.*, 1990; Focan *et al.*, 1990). Seven of these, actually addressed the value of standard, or almost standard, doses of chemotherapy compared with low doses (Tannock *et al.*, 1988; Habeshaw *et al.*, 1991; Carmo-Peirera *et al.*, 1987; Hoogstraten *et al.*, 1976; O'Bryan *et al.*, 1977; Forastiere *et al.*, 1982; Focan *et al.*, 1990). All but one highlighted an advantage in terms of response rate in standard dose arms, but none of these assessed the true role of dose intensity. In fact, analysing data from three studies with more than 100 patients each, two (Tannock *et al.*, 1988; Focan *et al.*, 1990) did not report any data about total dose delivered, while in the third (Habeshaw *et al.*, 1991), the significant increase in median dose intensity reported was associated with a significant increase in median total dose. A study conducted at the M.D. Anderson Clinic (Hortobagyi *et al.*, 1987), in which patients were randomised to receive a standard FAC or an escalated FAC (fluorouracil, adriamycin, cyclophosphamide) utilising a protected environment, failed to detect any difference either in response rate or in survival. This study is generally claimed as an example of failure of dose intensity, but it errs for the above-mentioned reason: the median dose, expressed in $mg\,m^{-2}$ per cycle, was different in the two groups. Moreover, a subsequent analysis by the same authors (Hortobagyi *et al.*, 1989) showed no significant difference in the dose intensity actually delivered, at 24 weeks, between high and standard dose arms. Indeed, it is not possible to assess the importance of cumulative dose independently of dose intensity. Experimental results (Skipper, 1990) indicate that cumulative dose and dose intensity may have independent and different effects. Therefore, to test the role of dose intensity it is important that planned and actual total administered dose are the same in both the control and the experimental groups of the study. This criterion was met in our study, in which patients in both groups received the same cumulative dose of chemotherapy and the same numbers of cycles.

The moderate increase in dose intensity obtained by accelerating CEF chemotherapy yielded an increase in response rate of borderline statistical significance. In addition, this response rate was higher than that obtained in two prior consecutive randomised trials carried out in our Institute (Conte *et al.*, 1987; 1990). When adjusted for prognostic factors, treatment retained its significant effect on response rate. Noteworthy was the fact that the effect of the dose intensity could be analysed without other confounding factors such as schedule, cumulative dose or drugs utilised. However, since anti-tumour response was not the primary objective of the study and given the small sample size, this result should be considered with caution.

In conclusion, the present study demonstrates that, with GM-CSF support, acceleration of the CEF regimen can be accomplished, achieving an almost 30% increase of dose intensity over the standard schedule. CEF acceleration is feasible and haematological toxicity is manageable for at least six courses in an outpatient setting.

We thank Laura Stelé and Monica Guelfi for careful data managing.
This study was supported jointly by Schering-Plough Corporation, AIRC grant and CNR Grant No. 93.02275-PF39 (ACRO).

### References

AM JOINT COMMITTEE ON CANCER (1992). *Breast Manual for Staging of Cancer*, 4th edn, pp. 149–154. Lippincott: Philadelphia.

ARDIZZONI, A., SERTOLI, M.R., CORCIONE, A., PENNUCCI, M.C., BALDINI, E., INTRA, E., FERRARINI, M., ROSSO, R., MAZZANTI, P. & PISTOIA, V. (1990). Accelerated chemotherapy with or without GM-CSF for small cell lung cancer: a non-randomised trial. *Eur. J. Cancer*, **26**, 937–941.

ARDIZZONI, A., VENTURINI, M., CRINO, L., SERTOLI, M.R., BRUZZI, P., PENNUCCI, M.C., MARIANI, G.L., GARRONE, O., BRACARDA, S., ROSSO, R. & VAN ZANDWIJK, N. (1993). High dose-intensity chemotherapy, with acclerated cyclophosphamide–doxorubucin–etoposide and granulocyte–macrophage colony stimulating factor, in the treatment of small cell lung cancer. *Eur. J. Cancer*, **29**, 687–692.

BECHER, R., WANDL, U., KLOKE, O., KURSCHEL, E., REINERS, C.H., BOEDDECKER, I., HIRCHE, H., ILLIGER, H.J., HARTWICH, G., WOLF, E. & SCHMIDT, C.G. (1990). Randomised study of different doses of epirubicin and identical doses of cylcophosphamide in advanced breast cancer (abstract 176). *Proc. Am. Soc. Clin. Oncol.*, **9**, 46.

BERETTA, G., TABIADON, D., TEDESCHI, L., GAMBROSIER, P., BERETTA, G.D. & LUPORINI, G. (1986). Front line treatment with CMF variations for advanced breast carcinomas. A randomised study (abstract 300). *Proc. Am. Soc. Clin. Oncol.*, **5**.

BONADONNA, G. & VALAGUSSA, P. (1981). Dose–response effect of adjuvant chemotherapy in breast cancer. *N. Engl. J. Med.*, **304**, 10–15.

BRONCHUD, M.H., HOWELL, A., CROWTHER, D., HOPWOOD, P., SOUZA, L. & DEXTER, T.M. (1989). The use of granulocyte-colony stimulating factor to increase the intensity of treatment with doxorubicin in patients with advanced breast and ovarian cancer. *Br. J. Cancer*, **60**, 121–125.

CARMO-PEREIRA, J., COSTA, F.O., HENRIQUES, E., GODINHO, F., CANTINHO-LOPES, SALES LUIS, A. & RUBENS, R.D. (1987). A comparison of two doses of adriamycin in the primary chemotherapy of disseminated breast carcinomas. *Br. J. Cancer*, **56**, 471–473.

CONTE, P.F., PRONZATO, P., RUBAGOTTI, ALAMA, A., AMADORI, D., DEMICHELI, R., GARDIN, G., GENTILINI, P., JACONUZZI, A., LIONETTO, R., MONZEGLIO, G., NICOLIN, A., ROSSO, R., SISMONDI, P., SUSSIO, M. & SANTI, L. (1987). Conventional versus cytokinetic polychemotherapy with estrogenic recruitment in metastatic breast cancer: results of a randomised cooperative trial. *J. Clin. Oncol.*, **5**, 339-347.

CONTE, P.F., GARDIN, G., MIGLIETTA, L., PACE, M., PRONZATO, P., ROSSO, R., RUBAGOTTI, A., SPECIALE, A., AMADORI, D., MOSSETTI, C., CARNINO, F. & MONZEGLIO, G. (1990). Chemotherapy with estrogenic recruitment in locally advanced (LABC) and metastatic breast cancer: results of two randomised trial (abstract 162). *Proc. Am. Soc. Clin. Oncol.*, **9**, 43.

CRAWFORD, J., OZE, H., STOLLER, R., JOHNSON, D., LYMAN, G., DABBARA, I., KRIS, M., GROUS, J., PICOZZI, V., RAUSCH, G., SMITH, R., GRADISHAR, W., YAHANDA, A., VINCENT, M., STEWART, M. & GLASPY, J. (1991). Reduction by granulocyte colony-stimulating factor of fever and neutropenia induced by chemotherapy in patients with small cell lung cancer. *N. Engl. J. Med.*, **325**, 164-170.

DEMETRI, G.D. & ANTMAN, K.H.S. (1992). Granulocyte-macrophage colony stimulating factor (GM-CSF): preclinical and clinical investigations. *Semin. Oncol.*, **4**, 362-385.

EBBS, S.R., SAUNDERS, J.A., GRAHAM, H., A'HERN, R.P., BATES, T. & BAUM, M. (1989). Advanced breast cancer. A randomised trial of two different dosages and two administration systems. *Acta Oncol.*, **28**, 887-892.

FOCAN, C., CLOSON, M.T.H., ANDRIEN, J.M., DICATO, M., LOBELLE, J.P., LONGREE, L. & RIES, F. (1990). Dose response relationship with epirubicin as first line chemotherapy for advanced breast cancer. A randomised trial (abstract P3:7). *Ann. Oncol.*, **1**, 18.

FORASTIERE, A.A., HAKES, T.B., WITTES, J.T. & WITTES, R.E. (1982). Cisplatin in the treatment of metastatic breast carcinoma: a prospective randomised trial of two dosage schedule. *Am. J. Clin. Oncol.*, **5**, 243-247.

GABRILOVE, J.L., JAKUBOWSKI, A. & SCHER, H. (1988). Effect of granulocyte colony-stimulating factor on neutropenia and associated morbidity due to chemotherapy for transitional cell carcinoma of urothelium. *N. Engl. J. Med.*, **318**, 1414-1422.

HABESHAW, T., PAUL, J., JONES, R., STALLARD, S., STEWARD, M., KAYE, S.B., SOUKOP, M., SYMON, R.P., REED, N.S. & RANKIN, E.M. (1991). Epirubicin at two dose levels with prednisolone as treatment for advanced breast cancer: the results of a randomised trial. *J. Clin. Oncol.*, **9**, 295-304.

HAMM, J.T., SCHILLER, J.H., OKEN, M.M., GALLMEIER, W.H., RUSTHOVEN, J. & ISRAEL, R.J. (1993). Dose-ranging study on hrGM-CSF in small cell carcinoma. *J. Clin. Oncol.*, in press.

HENDERSON, I.C., HAYES, D.F. & GELMAN, R. (1988). Dose-response in the treatment of breast cancer: a critical review. *J. Clin. Oncol.*, **6**, 1501-1515.

HOEKMAN, K., WAGSTAFF, J., VAN GROENINGEN, C.J., VRMORKEN, J.B., BOVEN, E. & PINEDO, H.M. (1991). Effects of recombinant human granulocyte-macrophage colony stimulating factor on myelosuppression induced by multiple cycles of high-dose chemotherapy in patients with advanced breast cancer. *J. Natl Cancer Inst.*, **83**, 1546-1553.

HOOGSTRATEN, B., GEORGE, S., SAMAL, B., RIVKIN, S.E., COSTANZI, J.J., BONNET, J.D., THIGPEN, T. & BRAINE, H. (1976). Combination chemotherapy and adriamycin in patients with advanced breast cancer. *Cancer*, **38**, 13-20.

HORTOBAGYI, G.N., BODEY, G.P., BUZDAR, A.U., FRYE, D., LEGHE, S.S., MALIK, R., SMITH, T.L., BLUMENSCHEIN, G.R., YAP, H.Y. & RODRIGUEZ, V. (1987). Evaluation of high-dose versus standard FAC chemotherapy for advanced breast cancer in protected environment units: a prospective randomised study. *J. Clin. Oncol.*, **5**, 354-364.

HORTOBAGYI, G.N., HRYNIUK, W.H., FRYE, D., GREMM, J. & BUZDAR, A.U. (1989). Dose-intensity analysis of high-dose chemotherapy for metastatic breast cancer (abstract 1004). *Proc. Am. Assoc. Cancer Res.*, **30**, 253.

HRYNIUK, W. & BUSH, H. (1984). The importance of dose intensity in chemotherapy of metastatic breast cancer. *J. Clin. Oncol.*, **2**, 1281-1288.

KAPLAN, L.D., KAHN, J.O., CROWE, S., NORTHFELT, D., NEVILLE, P., GROSSBERG, H., ABRAMS, D.I., TRACEY, J., MILLS, J. & VALBERDING, P.A. (1991). Clinical and virologic effects of recombinant human granulocyte-macrophage colony stimulating factor in patients receiving chemotherapy for human immunodeficiency virus associated non-Hodgkin's lymphoma: results of a randomised trial. *J. Clin. Oncol.*, **6**, 929-940.

LIESCHKE, G.J., CEBON, J. & MORSTYN, G. (1989). Characterization of the clinical effects after the first dose of bacterially synthesized recombinant human granulocyte-macrophage colony stimulating factor. *Blood*, **74**, 2634-2643.

LOGOTHETIS, J.C., DEWEUS, F.H., SELLA, A., AMATO, R.J., KILBOURN, R.G., FINN, L. & GUTTERMAN, J.U. (1990). Escalated therapy for refractory urothelial tumours: methotrexate-vinblastine-doxorobucin-cisplatin plus unglycosylated recombinant human granulocyte-macrophage colony-stimulating factor. *J. Natl Cancer Inst.*, **82**, 668-672.

MILLER, A.B., HOOGSTRATEN, B. & STAQUET, M. (1981). Reporting results of cancer treatment. *Cancer*, **47**, 207-214.

O'BRYAN, R.M., BAKER, L.H., GOTTLIEB, J.E., RIVKIN, S.E., BALCERZAK, S.P., GRUMET, G.N., SALMON, S.E., MOON, T.E. & HOOGSTRATEN, B. (1977). Dose response evaluation of adriamycin in human neoplasia. *Cancer*, **39**, 1940-1948.

SKIPPER, E.H. (1990). Dose intensity versus total dose of chemotherapy: an experimental basis. In *Important Advances in Oncology 1990*, DeVita Jr, V.T., Hellman, S., Rosemberg, S.A. (eds). pp. 43-64. Lippincott: Philadelphia.

TANNOCK, I.F., BOYD, N.F., DEBOER, G., ERLICHMAN, C., FINE, S., LAROCQUE, G., MAYERS, C., PERRAULT, D. & SUTHERLAND, H. (1988). A randomised trial of two dose levels of cyclophosphamide, methotrexate and fluorouracil chemotherapy for patients with metastatic breast cancer. *J. Clin. Oncol.*, **6**, 1377-1387.

TRILLET-LENOIR, V., GREEN, J., MANEGOLD, C., VON PAWEL, J., GATZMEIER, V., LEBEAU, B., DEPIERRE, A., JOHNSON, P., DECOSTER, G., TOMITA, D. & EWEN, C. (1993). Recombinant granulocyte colony stimulating factor reduces the infectious complications of cytotoxic chemotherapy. *Eur. J. Cancer*, **3**, 319-324.